# NKT10 cells: a novel *i*NKT cell subset

**DOI:** 10.18632/oncotarget.5270

**Published:** 2015-08-26

**Authors:** Gerhard Wingender, Duygu Sag, Mitchell Kronenberg

**Affiliations:** Izmir Biomedicine and Genome Center (iBG-izmir), Dokuz Eylul University Health Campus, Balcova/Izmir, Turkey, and La Jolla Institute for Allergy and Immunology (LJI), La Jolla, CA, USA

**Keywords:** *i*NKT cells, innate T cells, NKT10 cells, IL-10

Invariant Natural Killer T (*i*NKT) cells are a unique subset of T cells that combine features of innate NK cells and of adaptive memory T cells [1, 2]. This hybrid character of *i*NKT cells, combined with the fact that they express an invariant TCR α-chain, led to their naming. In contrast to conventional T cells, *i*NKT cells recognize glyolipid antigens presented to them by the non-polymorphic MHC class I-homologue CD1d. The best-studied antigen for *i*NKT cells is α-galactosylceramide (αGalCer), which is the chemically optimized version of a naturally occurring antigen, presumably from bacteria. Following TCR stimulation *i*NKT cells rapidly produce copious amounts of various cytokines, including Th1-, Th2-, and Th17 cytokines. Through these cytokines *i*NKT cells can have a pronounced effect on the immune system, impacting an impressive variety of different immune reactions. They are involved in a range of chronic and acute inflammatory processes, including responses to pathogens and tumors, as well as in autoimmune responses. Furthermore, the antigenic response of *i*NKT cells is highly conserved in evolution, with mouse *i*NKT cells able to recognize human CD1d and *vice versa*. As basically all human *i*NKT cells share the same invariant TCR and respond to the same CD1d/antigen complexes in a comparable fashion, their therapeutic potential is great. Therefore, it is not surprising that αGalCer and other *i*NKT cell antigens are under continuing development as therapeutic agents [1, 2].

Although essentially all *i*NKT cells recognize αGalCer bound to CD1d via their invariant TCR, it became clear in recent years that *i*NKT cells are not uniform [1, 2]. Based on preference in the production of particular cytokines and the expression of specific transcription factors, *i*NKT cells can be subdivided into subsets that are reminiscent to CD4^+^ T helper cell subsets. In particular, NKT1, NKT2 and NKT17 cells are biased to the production of Th1, Th2 and Th17 cytokines, respectively. These subsets develop naturally and can be detected in the thymus. Additional subsets were found to differentiate following antigenic exposure: T_FH_-like NKT_FH_ cells and *i*NKT cells expressing FoxP3 [1, 2].

Recently, we described a novel *i*NKT cell subset with IL-10-dependent regulatory function, which we termed NKT10 cells [3]. Interestingly, NKT10 cells express several markers commonly associated with regulatory T cells, including CD152 (CTLA4), CD279 (PD1) and CD304 (Neuropilin-1). Following antigenic stimulation the production of pro-inflammatory cytokines by NKT10 cells was greatly reduced compared to other *i*NKT cell subsets. Importantly, however, NKT10 cells were able to produce IL-10. Through this IL-10 production, NKT10 cells could impair anti-tumor immune responses and protect mice against experimental autoimmune encephalomyelitis (EAE), a mouse model of autoimmune disease [3]. Although, production of IL-10 by *i*NKT cells had been reported previously (see [3] for full discussion and references), a separate population dedicated to producing IL-10 with a distinct phenotype had not been described. In mice NKT10 cells were found in the thymus, spleen and other organs, but they were particularly frequent in white adipose tissue, where up to 13.5% of the *i*NKT cells produced IL-10 following activation [3]. Furthermore, we could detect NKT10 cells in human peripheral blood mononuclear cells, albeit at a low frequency (0.5%) [3]. Subsequently, others demonstrated that adipose NKT10 cells are important in maintaining the anti-inflammatory environment in the adipose tissue. In a mouse model, the presence of NKT10 cells supported the expansion of regulatory T cells and anti-inflammatory M2 macrophages [4].

Importantly, we also noted that NKT10 cells could be expanded greatly by *in vivo* treatment of mice with αGalCer [3]. The reduced production of pro-inflammatory cytokines by αGalCer-expanded NKT10 cells has previously led researchers to the erroneous conclusion that αGalCer stimulation of *i*NKT cells would induce an inactive state resembling anergy. Interestingly, the expansion of NKT10 cells by αGalCer was not a default response to strong antigenic stimulation. Although presentation of αGalCer by a bone marrow derived cell type could induce expansion of NKT10 cells, presentation by either DCs or B cells was not required [5], suggesting either redundancy or that this requires so far unknown properties of the cell presenting αGalCer.

The stimulation of *i*NKT cells with αGalCer is characterized by a mixed Th0-response, with both IFNγ and IL-4 being produced. Some other *i*NKT cells can alter the composition of cytokines measured in the plasma towards either IFNγ (Th1-biasing antigens) or IL-4 (Th2-biasing). This ability allows *i*NKT cells to orchestrate the ensuing global immune response towards either a Th1- or Th2-type. Importantly, the Th1/Th2-biasing nature of the antigen depends on the *trans*-activation of NK cells down-stream of *i*NKT cell activation, as the immediate response of *i*NKT cells towards antigenic stimulation is always a mixed Th0-response [6]. We have found that the ability of αGalCer to expand NKT10 cells *in vivo* was shared with four Th1-biasing antigens with related structures (C-Gly, EF77, SMC124 and DB06-1) [5, 7]. In contrast, a Th2-biasing *i*NKT cell antigen (OCH), or cytokine-driven *i*NKT cell activation due to TLR engagement or infections, did not induce NKT10 cell expansion [5].

NKT10 cells represent the first *i*NKT cell subset with a regulatory function under resting, steady-state conditions in mice and humans. We expect that this knowledge and the selective activation of particular *i*NKT cell functional subsets will help to resolve current controversies about the dichotomous nature of *i*NKT cells observed in various studies, namely their ability to exert either pro- or anti-inflammatory effects. Together with our data on the antigenic requirements for NKT10 cell expansion *in vivo*, such knowledge will likely help to understand the functional consequences of glycolipid antigens in therapy and how to deliberately tailor *i*NKT cell responses for therapeutic applications.
